# Hearing the patient voice for persistent pain intervention development: recommendations for using a bespoke online discussion forum for qualitative data collection

**DOI:** 10.1177/20494637241254098

**Published:** 2024-07-23

**Authors:** Charlotte Woodcock, Nicola Cornwall, Sarah A Harrisson, Clare Jinks, Alison Buttery, Julie Ashworth, Christian D Mallen, Lisa Dikomitis, on behalf of the PROMPPT team

**Affiliations:** 1 Centre for Musculoskeletal Health Research, School of Medicine, 4212 Keele University, Staffordshire, UK; 2 Midlands Partnership University NHS Foundation Trust, Haywood Hospital, Staffordshire, UK; 3 4212 Keele University, Staffordshire, UK; 4 Centre for Health Services Studies and Kent and Medway Medical School, 152160 University of Kent, Canterbury, UK

**Keywords:** Chronic pain, intervention development, netnography, online methods, primary care, person-based approach, patient and public involvement, qualitative methods

## Abstract

**Introduction:**

Understanding patients’ experiences is important when developing interventions for people living with persistent pain. Interviews and focus groups are frequently used to capture beliefs, views, and perspectives. These methods often require a commitment to a predetermined date and time that may present a barrier to participation. An asynchronous online discussion forum, specifically designed for research purposes, provides an alternative and potentially more accessible method for participation. In this article we discuss a bespoke online discussion forum, the Q-PROMPPT blog, as a case example.

**Methods:**

We describe how we developed the Q-PROMPPT blog, with patient and public involvement, and its use as an innovative method for qualitative data collection in the context of developing an intervention for patients prescribed opioids for persistent pain. Drawing on our experiences we discuss the following areas: planning and design, participant recruitment and registration, and participant experience and engagement.

**Results:**

We identify and address key concerns for each area of the Q-PROMPPT blog: planning and design: choosing software, assigning roles, designing the interface to promote usability; recruitment of participants: recruiting eligible participants, participant anonymity; participant experience and engagement: mitigating risk of harm, facilitating discussions, planning for forum close.

**Conclusion:**

Based on our lessons learnt, we outline recommendations for using a bespoke online discussion forum as a qualitative method to inform intervention development for people living with persistent pain. These include collaboration with information communication technology teams, co-design with patient and public partners, minimising risk of imposter participants and developing trust and online community identity.

## Introduction

When developing interventions for people living with persistent non-cancer pain (hereafter referred to as ‘persistent pain’), researchers need to adopt processes for maximising an intervention’s potential effectiveness.^1^ Central to these processes is understanding the experiences, needs and preferences of intervention users.^2^ Qualitative research methods, such as interviews and focus groups, are frequently used to help gain this understanding.^3^ Relying on methods that ask for a commitment to a set time and place may, however, inadvertently exclude some participants. The unpredictable nature of persistent pain may make it hard for participants to attend a pre-arranged interview or focus group. Other challenges associated with persistent pain, such as stigma of the condition or with using certain medication (e.g. opioids), may also make in-person discussions around illness experiences difficult.^4^

An online discussion forum (hereafter referred to as ‘online forum’) is an asynchronous method that offers an alternative way for collecting qualitative data and can help overcome these barriers to participation. For people living with persistent pain, online forums have the potential to provide an accessible and appealing method for participating in research^5^ as they offer flexibility as to when and where participation occurs. Participants can log in at a time and place preferable to them with no requirement to travel.^6^ The asynchronous format also gives participants time and space to consider, reflect, and amend contributions without any pressure to respond in the limited timeframe of an interview or dynamic of a focus group.^7^

Conducting research using an online forum can also benefit researchers. It makes travel redundant, expands access to geographically dispersed or hidden populations, and online forum entries are automatically time-stamped and reliably attributed to participants.^7^ Online forums use unidentifiable usernames promoting participant anonymity and enhancing disclosure when researching sensitive or stigmatized topics^8^ to facilitate rich and insightful contributions. Data are immediately available to download and analyse^5,9^ meaning there is no need for transcription costs and potential inaccuracies (e.g. misheard utterances, mistaken attributions, misinterpretation of content).^10^

The structure of an online forum typically includes a homepage with a list of topics. Each topic has its own page that begins with an opening question followed by a discussion thread. A discussion thread (sometimes called a topic thread or threaded discussion) is a series of responses (referred to as posts) around a specific topic. Posts are often text-based, although many online forums support other forms of expression such as emoticons, images, audio, video, voting polls, and sharing of website links. Discussion threads allow for a conversation-style of discourse encouraging a natural flow of discussion. Each post is prefixed with basic participant information (e.g. @username, time published) and displayed in chronological order with the most recent post placed at the top of a page to avoid excessive scrolling. Participants can ‘tag’ each other by typing another’s @username. This action will alert the tagged participant to a specific post through an online forum’s notification system such as an automatically generated email.

The use of online methods has proliferated post COVID-19 and it is important to continually appraise the strengths and limitations of these methods alongside considerations for optimising use for inclusivity and generation of rich data.^11–13^ Although there is some guidance for using internet forums for data collection, these are mainly concerned with established online health communities.^14^ Less is known about the optimal development and management of ‘bespoke’ forums that are built and hosted solely for research purposes, despite explicit calls for researchers to share experiences on using online methods for their use in clinical research contexts.^13^

### Learning lessons from the ‘Q-PROMPPT blog’

Here, we draw upon our experiences of developing and using a bespoke online forum to collect data as part of a person-based approach^2^ to develop a new primary care pain review for patients prescribed long-term opioids for persistent pain (**P**roactive clinical **R**eview of patients taking **O**pioid **M**edicines long-term for persistent **P**ain led by clinical **P**harmacists in primary care **T**eams – the PROMPPT research programme). Our forum, called the Q-PROMPPT blog, aimed to explore experiences and views of people living with persistent pain of their: pain, pain medicines, pain management, and the proposed pain review. We describe key considerations and how we applied these to the Q-PROMPPT blog. We follow these by summarising our experiences and lessons learnt into recommendations for developing and using online forums as a qualitative method.

## Key considerations applied to the Q-PROMPPT blog

### Planning and design

#### Choosing software

Online forum software needs to be identified, set-up, and tested. Many software options exist,^15^ and considerations need to be made around cost, digital skills of researchers and participants, level of IT support required, customisation features, communication tools, usability on different devices, and access to data. We consulted with our institution’s information communication technology (ICT) department and chose Discourse (n.d.) for development of the Q-PROMPPT blog,^16^ an open-source software that was customisable to our research needs. Hosting Discourse on our institution’s servers avoided a nominal monthly hosting fee, and ensured data and participant information were stored securely and not managed by a third party. Discourse supported a responsive layout that adapts to different device layouts (e.g. desktop, tablet, smartphone) and has inbuilt analytics that allows basic monitoring of participant activity (e.g. date and time logging on, length of time per online forum visit, number of comments posted) that may be useful to measure participant engagement. We found continued ICT support useful for software set-up, provide basic software training, and on-going support to address any unforeseen technical issues.

#### Assigning roles

Researchers and study participants will need online forum accounts with usernames and passwords to log in to the software platform. Accounts can be assigned different roles that determine levels of access. The exact permissions available may vary across different software. At a minimum, participants need access to read and submit posts to discussion threads, and, in some cases, have permission to create new topic pages for discussion. The research team will likely have additional permissions depending on their role in developing and implementing the online forum (see Table 1).

**Table 1.** Discussion forum roles and permissions.

Role	Permissions1								
	Submit a post	Edit own post	Edit others’ posts	Flag posts	Process flagged posts	Create topic pages	Delete topic pages	Change others’ permissions	Access participant information
Administrator	Yes	Yes	Yes	Yes	Yes	Yes	Yes	Yes	Yes
Moderator 2	Yes	Yes	Yes	Yes	Yes	Yes	Yes	No	No
Facilitator 2	Yes	Yes	Yes	Yes	Yes	Yes	Yes	No	No
Participant	Yes	Yes	No	Yes	No	Sometimes	No	No	No

*Note 1.* What permissions are available will depend on the options provided by the discussion forum software.

*Note 2.* The role of moderator and facilitator can be separated between two researchers or subsumed into a single role.

For the Q-PROMPPT blog, members of the research team were assigned moderator and facilitator roles that gave user permissions to effectively manage the online forum. As participant posts were immediately available to view online, frequent moderation was necessary. Five moderation slots were timetabled in any 24-h period, meaning daily moderation occurred at least every 4 h from 8 a.m. to 10 p.m., with a maximum 10-h gap overnight. We therefore deemed it necessary to have a team of moderators (*n* = 5) to ensure adherence to our community guidelines informed by ethical guidelines for internet-mediated research^17^ (see Supplemental File 1). We found separating moderator and facilitator roles allowed one person to focus on facilitation and immerse themselves in the data, whilst moderators could share responsibility of checking the appropriateness of posts at regular intervals on a daily basis. Preparation for each role required approximately 2 hours training.

Research team members did not have access to identifiable participant information. We hoped distancing our ability to know anything identifiable about participants would help minimise power-differentials between researchers and participants and build trust. We do acknowledge, however, that a researcher’s position of power can still be created through the academic language used in study materials, institutional support of a project, researcher-selected discussion topics, and sharing of researcher titles.^18^ Members of the ICT department were given administrator roles to manage permissions of all other user accounts and had access to participant information (i.e. name and email) to be able to send study relevant information and contact participants if concerns arose around safeguarding and wellbeing.

#### Designing the interface to promote usability

Usability testing prior to data collection helps identify potential user difficulties and technical issues, including trouble creating usernames and issues logging on to an online forum, all of which could negatively impact participant engagement with the research.^12^ To explore the usability of the Q-PROMPPT blog, we conducted pilot testing with six members of the research team. Testing highlighted that when an internet browser’s cookies are disabled it was not possible to register a new user account. Consequently, we included a section on the study website FAQs page for trouble-shooting potential issues around the online forum registration process.

We also conducted usability testing with four members from the PROMPPT research programme’s Patient and Public Research User Group (RUG) as part of a 3-h workshop. RUG members responded to worksheet prompts that required successful navigation of research processes including online consent, online forum registration, and posting to a topic’s discussion thread. This phase of testing suggested website hyperlinks to access the Q-PROMPPT blog information page were not easy to find. We subsequently changed the colour of these hyperlinks to make them stand out more to potential participants. Following these improvements, we carried out a final round of testing with remote users naive to the study. These users successfully navigated key research processes suggesting the Q-PROMPPT blog was technically sound, accessible and easy-to-use for participants.

It should be noted that the planning and design phase described took approximately 12 months to complete.

### Participant recruitment and registration

#### Recruiting eligible participants

Recruitment methods for online forums vary and can involve online advertising as well as conventional methods. We created social media accounts dedicated to the PROMPPT research programme 6-months in advance of study commencement. This was beneficial in building a community of interested people to target when recruiting to the Q-PROMPPT blog. We posted participant invitations on our social media channels on X (2024; formally Twitter)^19^ and Facebook (2024)^20^ to over 300 followers, as well as online pain support groups and charities. Aware potential participants may not regularly visit such online platforms, we also displayed study posters and flyers in primary care practices, community pain services and community pharmacies.

**Figure 1.**
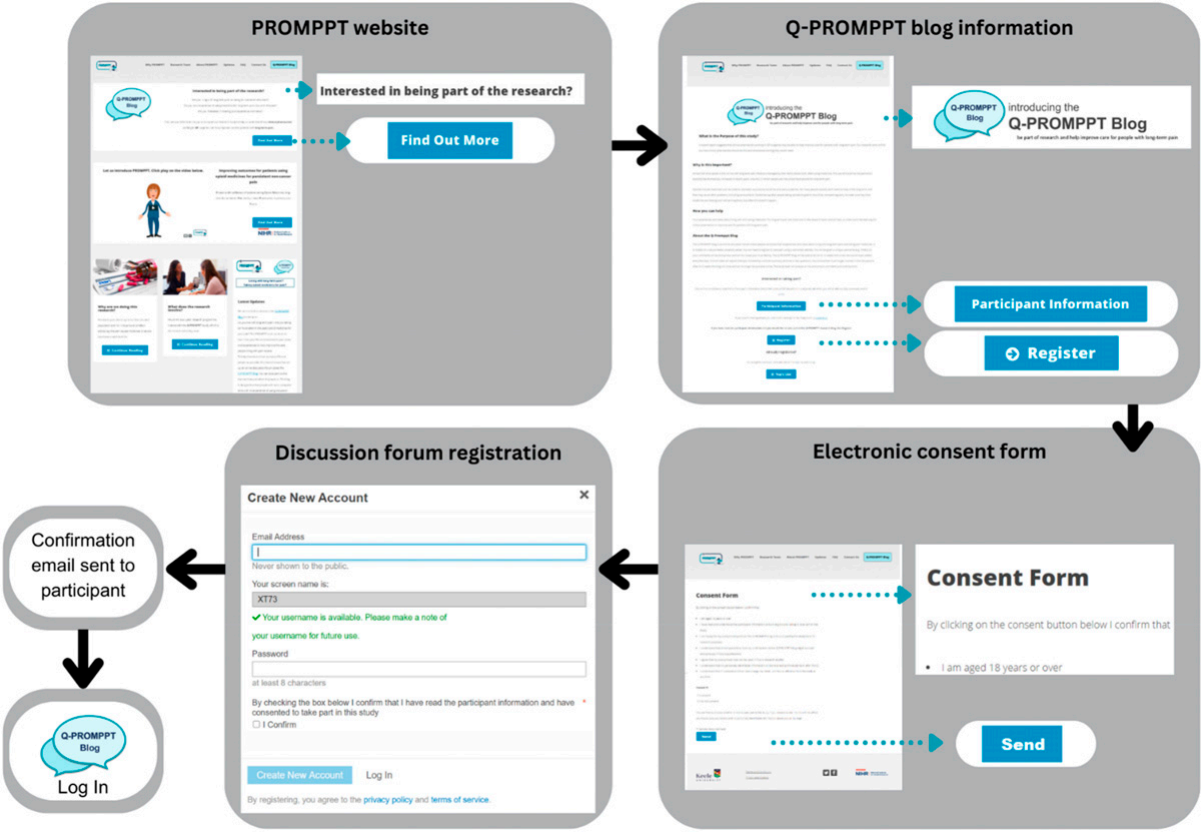
The participant recruitment pathway.

We wanted to remove potential barriers to recruitment and participant burden of having to contact the research team or complete surveys for sampling purposes prior to online forum registration. Instead, we relied on participants to self-select and assess their own eligibility for the study against advertised criteria (e.g. >18 years, living in the UK, with experience of taking long-term opioids for persistent pain). To reduce the risk of recruiting ineligible people, we directed recruitment efforts through persistent pain related charities, community groups, healthcare services and social media accounts registered in the UK to target people most likely to be eligible to participate.^21^ Recruitment advertisements directed people to the PROMPPT (2024) website containing a Q-PROMPPT Blog information page that contained links to a participant information sheet and electronic consent form. By completing consent, participants agreed they met study inclusion criteria. Following consent, participants submitted a registration form that generated an automatic confirmation email requiring a participant response to guard against registration-bots. Once confirmed, participants were able to log in to the Q-PROMPPT blog using their username and password (see Figure 1).

#### Participant anonymity

Online forums allow for anonymous usernames to be assigned to participants. Anonymity serves to minimise social influences that are more prevalent when research is conducted in-person. In face-to-face encounters participants may modify responses to support research goals, conform to perceived group norms, or be influenced by conscious and unconscious biases triggered by characteristics such as age, gender, socioeconomic status, professional status, sexual orientation, and ethnicity.^22^

To protect participants’ anonymity, we chose to assign participant usernames using unique codes.^8^ This was in contrast to previous research where online forum participants have either chosen whether or not to post comments anonymously^12^ or asked to create their own username.^23^ However, both of these approaches risk making participants identifiable through not choosing to post anonymously or creating a username that is similar to one used for other online accounts.^9,24^

To generate unique codes, members of our ICT department scripted a bespoke algorithm, using a combination of Javascript and Jquery, that created a unique combination of two letters and two numbers (e.g. WH37) during registration to the Q-PROMPPT blog. This approach was favoured over random word combinations that may unintentionally create inappropriate phrases. Prior to analysis, to further protect participant identity, these usernames were replaced with participant identification codes (e.g. WH37 replaced with code BP01).

### Participant experience and engagement

#### Mitigating risk of harm

Researchers have a responsibility to protect the safety of participants as well as themselves. A lack of physical cues can make it difficult to identify when participants are distressed and such risks need to be identified and mitigation measures put in place.^9,17^ To help minimise possibility of distress, online forums have community guidelines that set out appropriate online behaviour and information on how to seek further support if needed. The Q-PROMPPT blog community guidelines aimed to promote a friendly and psychologically safe online environment by requesting participants to: (i) maintain anonymity, (ii) respect others, and (iii) stay on-topic. Posts perceived to contain information in breach of these guidelines could be edited by our moderators. Participants also had the ability to ‘flag’ posts deemed inappropriate to bring these to the attention of the research team. We also published contact details of support agencies on the Q-PROMPPT blog FAQs page with hyperlinks to relevant organisations (e.g. Pain Concern, n. d.)^25^ and helpline numbers.

We also created a process for the possibility of participants becoming distressed. In the event of perceived participant distress, a message would be sent via the online forum’s private messaging system (copied to the participant’s email address) to offer an opportunity to discuss any concerns. However, we did not perceive a need to action this during the lifetime of the Q-PROMPPT blog.

#### Facilitating discussions

An online forum’s facilitator will regularly read participant posts and pose questions, provide prompts and probes, and check for understanding in a way that promotes participant discussion and on-going engagement in evolving and new discussion topics over time. We felt it was important to have a qualitative researcher, with experience of in-person focus groups and interviews, to adopt the facilitator role (CW) supported by a senior qualitative researcher (LD). To prepare for online facilitation, CW engaged with the extant literature regarding building online communities and participant facilitation, as well as piloting facilitation strategies during usability testing.^12,26^

One challenge for facilitators, which we also faced, is to engage a majority of participants in topic discussion threads. Typically, only a minority of participants will post frequently and others occasionally. Many will be read-only participants (sometimes referred to as ‘lurkers’) who do not contribute to discussions.^12^ Strategies used to promote engagement can include the use of monetary incentives.^12^ However, online methods that incentivise participation are at risk of attracting ‘imposter participants’ who misrepresent themselves as meeting a study’s inclusion criteria in order to receive payment associated with participation.^27^

We acknowledge that the Q-PROMPPT Blog was at risk of imposter participants as recruitment processes relied on participants’ identifying their eligibility against inclusion criteria. We did not offer financial incentives. We mitigated the risk of imposter participants through actively promoting engagement by communicating the value participants bring to the online forum.^26^ One strategy we used was to establish a community identity to develop participant commitment and engagement. We drew on social identity theory that proposes people are more likely to join and commit to a group based on the extent they share a group’s common interests, value community aims, and perceive community membership important for achieving goals they cannot accomplish alone.^28^ To highlight common interests (and therefore membership of the same social category), the first topic for discussion focused on an experience participants shared of living with persistent pain. To help participants understand and value the aims of the discussion forum, a statement that clearly defined the purpose of the research study was visible on the welcome page (*be part of research and help improve care for people with long-term pain*). The statement summarised a community goal likely to be valued by participants yet challenging to pursue at an individual level to help foster social cohesion.^28^

**Figure 2.**
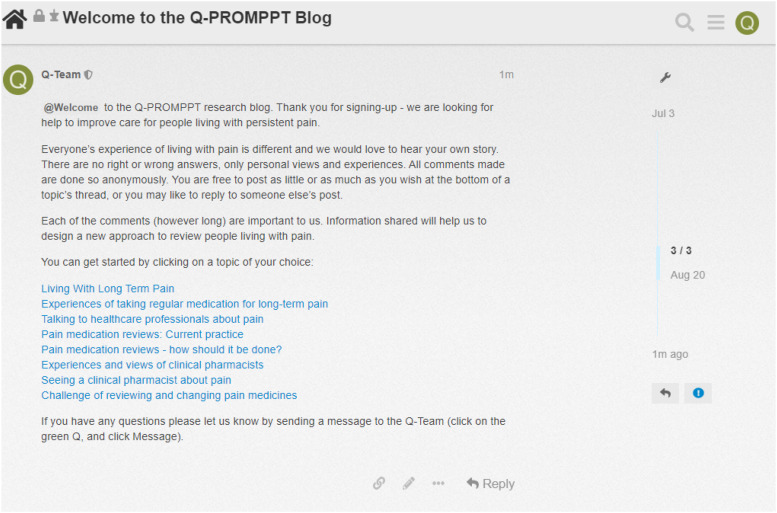
A welcome message posted to the Q-PROMPPT blog tagging the group @Welcome that included all read-only participants.

**Figure 3.**
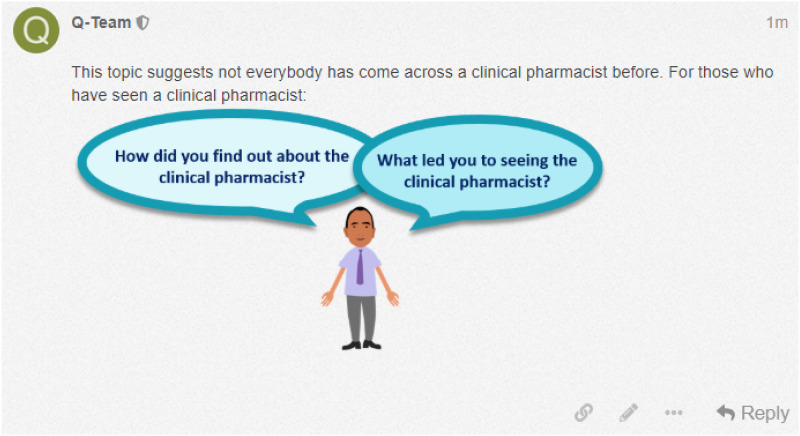
An example of a facilitator post using an image of a research avatar.

We also incorporated other strategies to further promote participant engagement, including: (i) starting topic-page discussions by posting an initial overview and starting question using video-animation and text (see Supplemental File 2); (ii) building trust between researchers and participants by welcoming new participants, posting timely responses, expressing empathy and understanding, acknowledging concerns and validating experiences, and thanking participants for their contributions; (iii) guiding and promoting discussion by posting prompts and probes, seeking clarification, summarising key points in discussion threads, and tagging participant @username to alert participants to new posts; as well as (iv) encouraging new voices to be heard by tagging read-only participants to a welcome message to encourage contributions to discussion threads (see Figure 2). To avoid facilitator posts becoming lost and unnoticed in a busy discussion thread, we included an image of a researcher avatar to help these stand out against other text-only posts (see Figure 3).

#### Planning for closure of the online forum

Online forums can offer participants informational and social support,^26^ and participants may want to continue discussions after the research is complete.^12^ It is important to manage expectations around the purpose of the online forum and whether it is limited to research study objectives. A bespoke forum created for research purposes is likely to close once the research study is complete, however, some online communities may continue life beyond the initial research (e.g. Wallace et al., 2018).^29^

We managed participants’ expectations of the longevity of the Q-PROMPPT blog through the participant information sheet that told participants the online forum would close 10–12 weeks after it opened. A specific closing date was not determined to allow for a degree of flexibility for any active discussions to continue. Once an end date was decided, an administrator from the ICT department emailed all participants 1-week before the online forum was taken off-line. We also posted a 5-day countdown to closure on our social media networks and study website.

### Final reflections

Following ethics approval from East of England – Cambridge East research ethics committee, the Q-PROMPPT blog was available online for 77 days from 3^rd^ October 2019 to 18^th^ December 2019. During this time, 78 participants registered an anonymous username, and 69 participants went on to confirm their email address and log on between 1 and 30 times. Of these participants, 31 (44.93%) posted between 1 and 19 times to one or more discussion topics, totalling 160 comments altogether ranging in length from 19 to 2143 words. None of these participants were identified as ‘imposters’. From our experience, a bespoke online discussion forum provided an innovative and alternative method for people living with persistent pain. Data collected was integral to our development of an acceptable primary care pain review.^30^ Feedback from participants suggest they enjoyed being part of research and found the Q-PROMPPT blog to be a good way to share experiences. Our experience highlights a number of key considerations and what worked well when developing and using a bespoke online discussion forum. We summarise our recommendations for using an online forum for qualitative data collection in Table 2.

**Table 2.** Summary of key considerations, main concerns, and recommendations when using a bespoke online discussion forum.

	Key considerations	Main concerns	Recommendations
Planning and design	1. Choosing software	• Digital skills of researchers and participants• Secure storage of data	• Collaborate with information communication technology (ICT) department to help identify appropriate software and secure hosting on institution servers
			• Secure ICT support for the duration an online forum is active to address any unforeseen technical issues
			• Allow for at least 12-month development time from identifying what software to use to the online forum going live (i.e. commencement of data collection)
	2. Assigning roles	• Appropriate access and management of the forum	• Define and differentiate moderator and facilitator roles
			• Assign the role of forum administrator to an individual outside the immediate research team (e.g. ICT department) to maintain participant anonymity
	3. Designing the interface to promote usability	• Digital skills of researchers and participants	• Co-design the forum with patient and public partners, including usability testing, to develop an accessible and acceptable online forum
		• Usability of forum	• Include different modes of communication on the online forum to meet diverse needs (e.g. videos as well as text)
			• Include a ‘how-to’ video demonstrating how participants can communicate and contribute to topic discussion threads
Participant recruitment and registration	4. Recruiting eligible participants	• Reaching a geographically dispersed participant group	• Advertise online and off-line
		• Imposter participants	• Refrain from offering monetary incentives to avoid attracting disingenuous imposter participants, however, if reimbursement is offered, use BACS payments as opposed to vouchers^27^
			• Incorporate processes to check participant eligibility prior to registration to the online discussion forum (e.g. contact a member of the research team)
	5. Participant anonymity	• Participants disclose identifiable information	• Assign anonymous usernames
			• Prior to analysis, replace anonymous usernames with participant identification codes
			• Regularly moderate the online forum and remove potentially identifiable information (e.g. names, locations)
Participant experience and engagement	6. Mitigating risk of harm	• Inappropriate online forum behaviour	• Publish community guidelines outlining appropriate behaviour on the online forum
		• Lack of physical cues of participant distress	• Publish information of persistent pain support organisations and charities
			• Regularly moderate the online forum to identify posts that signify participant distress
			• Develop a protocol for responding to participant distress
	7. Facilitating discussions	• Prevalence of ‘read-only’ participants	• Develop online trust and a community identity
		• Participant disengagement over time	• Begin discussion threads with a topic’s description and question
		• Facilitator prompts and probes not seen in long discussion threads	• Respond promptly to participant posts
			• Make facilitator posts noticeable (e.g. by adding an image of a researcher avatar)
			• Tag participant usernames into online forum posts
			• Consider incentives for posting to the online forum (note the risk of attracting imposter participants)
	8. Planning for forum close	• Participants expecting an on-going forum	• Manage participants’ expectations of how long the online forum will be active
			• Inform participants when the online forum will close (e.g. via the participant information leaflet, email, social media, website, online forum Q&A page)

We acknowledge that online forums have the potential to reach underserved population groups, such as people taking opioids long-term for persistent pain, and encourage participation in research for people who do not want to take part in-person. However, the effectiveness of online forums in achieving this wider participation still needs to be assessed through collection of participant demographic data to describe and assess effectiveness, where this does not undermine participants’ trust.

## Supplemental Material

Supplemental Material - Hearing the patient voice for persistent pain intervention development: recommendations for using a bespoke online discussion forum for qualitative data collection Supplemental Material for Hearing the patient voice for persistent pain intervention development: recommendations for using a bespoke online discussion forum for qualitative data collection by Charlotte Woodcock, Nicola Cornwall, Sarah A Harrisson, Clare Jinks, Alison Buttery, Julie Ashworth, Christian D Mallen, Lisa Dikomitis and on behalf of the PROMPPT team in British Journal of Pain

**Figure:** Video 1.
