# Supplementary material for: Hearing the patient voice for persistent pain intervention development: recommendations for using a bespoke online discussion forum for qualitative data collection
Source: Br J Pain. 2024 Jul 23;18(6):461–71. doi: 10.1177/20494637241254098 (PMC11631626; doi:10.1177/20494637241254098)
Supplement: Supplemental Material - Hearing the patient voice for persistent pain intervention development: recommendations for using a bespoke online discussion forum for qualitative data collection [file sj-pdf-1-bjp-10.1177_20494637241254098.pdf]

Do you want live notifications when people reply to your posts? Enable Notifications .

Community Guidelines

Q-Team 5 Jul '19

The aim of the Q-PROMPPT blog is to provide a place where experiences and views can be shared in an environment which is friendly, respectful and inclusive. All participants should feel safe to anonymously contribute as little or as much as they wish to discussion topics. To help achieve this when posting comments please observe the following guidelines:

Maintain anonymity

- Please do not post personally identifiable information about yourself or others. Personally identifiable information includes:
  - Names (yours or others)
  - Addresses
  - GP practices or hospital names

Respect others

- Please respect other users
- Please keep it clean. Do not post any language that may be considered obscene or sexually explicit
- Please do not use racist, sexist, homophobic or other forms of hate speech or comments that could be interpreted as such
- Comments should not be malicious, inflammatory or offensive in nature
- Comments should not be obviously commercial, spam-like or trolling

Stay on-topic

- Each topic as a different title. Please try to continue the conversation or ask a question relating to the topic's title.
- Comments that are deemed completely off-topic will be moved to another area or removed.
- Please do not make any commercial endorsement or promotion of any product, service, or publication.

If you see a problem, flag it

The Q-Team will be working behind the scenes to ensure posts follow community guidelines. Posts not adhering to these guidelines will be edited or deleted by a moderator. If you notice any posts that may cause offence or concern please flag this to the attention of the Q-Team by clicking on the flag icon located underneath the post.

Medical guidance

It is not the aim of this site to provide guidance or recommendations relating to long-term pain. The views expressed are those of study participants and not necessarily those of the study team, Keele University, or NIHR. Advice offered by users of the site is not endorsed by the study team or Keele University, or NIHR.

If you need further medical guidance about your pain, or you are thinking of changing your medications, please consult your GP.

Reply

created Jul '19 | last reply Jul '19 | 1 reply | 56 views | 2 users

CLOSED JUL 3, '19

Bookmark Share Flag Reply

Normal You will be notified if someone mentions your @name or replies to you.

View Topics

This website presents independent research funded by the National Institute for Health Research (NIHR) under its Programme Grants for Applied Research Programme (Reference number RP-PG- 0617-20005).

The views expressed are those of the author(s) and not necessarily those of the NIHR or the Department of Health and Social Care.

© 2019. Keele University. All rights reserved.
